# Self‐esteem development during the transition to work: A 14‐year longitudinal study from adolescence to young adulthood

**DOI:** 10.1111/jopy.12713

**Published:** 2022-03-23

**Authors:** Lorenzo Filosa, Guido Alessandri, Richard W. Robins, Concetta Pastorelli

**Affiliations:** ^1^ Department of Psychology Sapienza University of Rome Rome Italy; ^2^ Department of Psychology University of California, Davis Davis California USA

**Keywords:** life events, maturity principle, personality development, school‐to‐work transition, self‐esteem development, social investment principle

## Abstract

**Introduction:**

Previous studies examined the trajectory of self‐esteem during critical developmental periods and over the life‐span. However, little is known about how self‐esteem changes during the school‐to‐work transition.

**Method:**

We examined the effect of beginning a job for the first time on self‐esteem development, using data from 368 adolescents assessed up to six times across a 14‐year time span. Specifically, we analyzed the pattern of self‐esteem change during the transition to work and whether the self‐esteem trajectory varied as a function of several school‐ and job‐related variables, while controlling for important covariates.

**Results:**

Results revealed linear increases in self‐esteem across the 14‐year study period, with partial support that the rate of increase slowed slightly after the school‐to‐work transition. We found significantly greater variability in the slopes after the transition, supporting the idea that people differ in the way they cope with the developmental tasks associated with important life transitions. We also found evidence for an interaction between college graduation and educational expectations, such that the positive effect of college graduation on self‐esteem change was stronger for those who graduated with low (vs. high) educational expectations.

**Conclusion:**

School‐to‐work transition has an effect on self‐esteem development. Developmental processes of findings were discussed.

## 1 INTRODUCTION

Global self‐esteem, an individual's subjective evaluation of his or her worth as a person (Donnellan et al., 2011), is one of the most widely studied constructs in psychology. Its popularity across different areas of psychology (i.e., personality, developmental, clinical and social psychology) is mainly due to its widespread implications for important life outcomes, such as school attainment, physical and mental health, psychosocial adjustment, and work success (Orth & Robins, In press). Given its beneficial consequences, researchers have focused on identifying factors that predict the development of self‐esteem.

Previous empirical studies have explored the normative trajectory of self‐esteem during important critical developmental periods, as well as over the entire life span (Orth et al., 2018). On average, self‐esteem shows linear increases across adolescence and young adulthood (Orth & Robins, 2019), although there is substantial individual variability in both the direction and magnitude of self‐esteem change during these developmental periods (Orth, 2017). This finding highlights the importance of identifying the sources of these individual differences, and thus the factors that can pull a youth's self‐esteem trajectory away from the normative (i.e., age specific) trajectory. Recently, researchers have pointed to life events, including starting a job for the first time, as among the most important factors shaping the development of self‐esteem from late adolescence to young adulthood (Bleidorn et al., 2018; Orth et al., 2018).

In fact, the school‐to‐work transition represents one of the first *turning points* individuals face in the early phase of their adult life course (Rutter, 1996; Schoon & Silbereisen, 2009). Formally, it can be defined as a “period and process in which young adults move from school and life as scholars and begin full‐time employment” (Saks, 2018; see also Morrison, 2002). This event entails important changes in the lives of youths because of (Allen & van de Vlier, 1984; Hogan & Roberts, 2004; Schoon & Silbereisen, 2009): (1) significant modifications in their daily routine and how their time is organized; (2) new tasks and responsibilities to deal with, because youths need to acquire new knowledge and skills to perform their job; (3) greater autonomy because youths are often personally responsible for their assigned tasks with less support from others compared to the school and home contexts; and (4) the formation of many new relationships because they need to meet and interact with coworkers, supervisors, subordinates, and/or clients and customers. According to social investment theory, these changes are likely to contribute to personality maturation in general, and positive self‐esteem development in particular, as youth adapt to the new challenges and opportunities they face in the workplace. In addition, after the completion of school, the workplace becomes the primary context in which most individuals experience achievement‐related successes and failure, which both theory and research suggests will impact self‐esteem development (Covington, 1992; Zheng et al., 2020). Moreover, based on sociometer theory (Leary, 2012; Leary & Baumeister, 2000), the substantial changes in one's social network that come with beginning a job are likely to lead to a new set of “significant others” (supervisors, coworkers, etc.) who might impact youths’ self‐esteem, either positively or negatively.

In the present study, we used data from a six‐wave longitudinal study of youths followed from age 13 to 31 to investigate changes in self‐esteem occurring as a result of youths’ transition into the job market. Similar to past research on self‐esteem change occurring during life transitions (Bleidorn et al., 2016; van Scheppingen et al., 2018), we assumed a discontinuous and multiphase perspective in conceptualizing change. Specifically, we examined the developmental trajectories occurring before and after the transition, as well as the possibility of an event‐induced sudden change occurring at the time of the transition. Furthermore, we tested whether the self‐esteem changes associated with the school‐to‐work transition were moderated by (1) youths’ educational expectations (i.e., how far they expected to go in school), (2) whether or not they graduated from college, (3) whether their educational expectations matched their educational attainment, (4) the age when the youth transitioned to work (i.e., the timing of the transition) and (5) the type of job (temporary vs. permanent).

### 1.1 Self‐esteem and role transitions

Global self‐esteem tends to show linear mean‐level increases from adolescence to young adulthood (Birkeland et al., 2012; Chung et al., 2014; Erol & Orth, 2011; Kiviruusu et al., 2015; Orth et al., 2018, 2010; Wagner et al., 2013; see Orth & Robins, 2019, for a review). Studies focused on domain‐specific self‐esteem (von Soest et al., 2016) or different populations of youths (i.e., African Americans and Latino youths; Erol & Orth, 2011; Zeiders et al., 2013) have provided similar results (but see Harris et al., 2018).

Theoretically, these results support two important principles of personality development (Orth et al., 2018). The *maturity* principle maintains that during the transition from adolescence to young adulthood, youths become more confident and emotionally stable as a consequence of becoming involved in new personal responsibilities and societal roles (Caspi et al., 2005; Donnellan et al., 2007; Roberts & Nickel, 2017). The *social investment* principle (Roberts & Nickel, 2017) states that engaging in adult societal roles requires youths to develop their independence and personal accountability in order to meet new expectations and demands (Leikas & Salmela‐Aro, 2015) because the adult roles imply a new status, social recognition, and social power (Dannefer, 1984; Gove et al., 1989; Trzesniewski et al., 2004, 2013). In short, changes in self‐evaluations emerge as a consequence of investing in these roles, with associated responsibilities representing an opportunity for success or failure (Orth et al., 2018; Roberts & Nickel, 2017).

Another important theoretical framework that can provide insight into the development of self‐esteem is sociometer theory (Leary, 2012; Leary & Baumeister, 2000; Orth & Robins, 2019; see Harris & Orth, 2020). Rooted in the assumption that social relationships are a key determinant of people's self‐esteem, sociometer theory suggests that self‐esteem levels provide a gauge of peoples’ social inclusion status (Leary, 2012; Leary & Baumeister, 2000). Accordingly, self‐esteem tends to increase when individuals’ experience greater social inclusion and acceptance, but decrease when individuals’ experience social exclusion and rejection. Importantly, sociometer theory maintains that social relationships are not the only source of self‐esteem, but rather any factor that “raises or lowers one's own relational value should influence self‐esteem” (Leary, 2012, p. 154). From this perspective, adult role transitions that entail new societal expectations, demands, and responsibilities may increase self‐esteem by bolstering young adults’ general sense of acceptance by others, but they may also lead to lower self‐esteem for individuals who do not adapt well to the transition and feel less included and accepted by others.

Extant research has demonstrated that life events involving role transitions have an impact on the self‐esteem trajectory during young adulthood (Orth et al., 2018). For example, youths starting a stable romantic relationship or marriage (Luciano & Orth, 2017; Wagner et al., 2015) often show an increase in self‐esteem, compared to those who remain single. The transition to parenthood is associated with a sudden decrease after childbirth (for mothers) and a gradual, long‐term decrease in self‐esteem during the years after childbirth, for both moms and dads (Bleidorn et al., 2016; van Scheppingen et al., 2018). Similarly, negative life events can lead to decreases in self‐esteem (Orth & Luciano, 2015). However, knowledge regarding the effect of youths’ first transition to work on self‐esteem is limited (Krauss & Orth, 2021), but to the extent that this transition promotes social status and acceptance we would expect based on sociometer theory and social investment theory that it would contribute to improvements in self‐esteem.

### 1.2 School‐to‐work transition and changes in self‐esteem

Starting one's first job brings about important changes in youths’ daily organization and tasks (Havingurst, 1972; Hutteman et al., 2014), including increases in responsibilities and demands (Saks, 2018; Schoon & Silbereisen, 2009). Successfully coping with new responsibilities requires detaching from past habits and goals to engage in new role‐assignments (Allen & van de Vlier, 1984). According to Nicholson (1984), this process affects personal habits, attitudes and social relationships, given the consequent changes in youths’ daily life environment. Most importantly, starting a job requires a specific learning process, namely organizational socialization (Bauer et al., 2007), that is necessary for acquiring the requisite knowledge and skills, internalizing organizational norms, gaining insight into the organizational culture, and learning how to fit in and be accepted by others in the new work environment (Alessandri et al., 2020; Ellis et al., 2015). Successfully undergoing such a process will likely lead to more positive feelings about one's worth as a person and one's social status within the organization.

**FIGURE 1:**
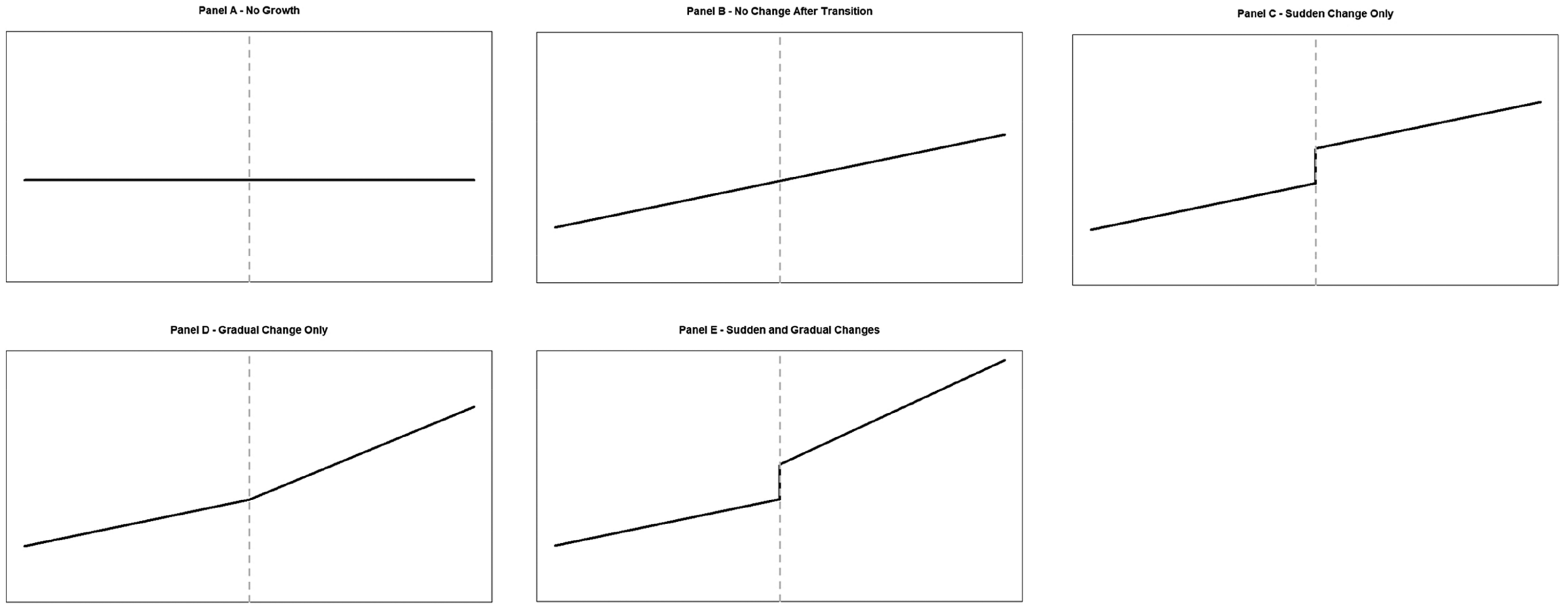
Theoretical models of self‐esteem change during the transition from school to work. The grey dotted vertical line represents the moment of transition to work. The solid lines represent changes in self‐esteem (adapted from Bleidorn et al., 2016; Doss et al., 2009)

Previous studies examining self‐esteem changes during the transition to adulthood have found an overall increase (Orth et al., 2018). For example, Chung et al. (2017) found that self‐esteem levels were relatively low during adolescence, but increased as youth transitioned into young adulthood. Wagner et al. (2013) also found a mean‐level increase during this period, but with large individual variability in the trajectory of self‐esteem. The only study that has specifically focused on self‐esteem changes following the transition to full‐time employment also reported increases. Specifically, Reitz et al. (2020), using a two‐wave longitudinal design, uncovered a small boost in self‐esteem after youths graduated from university and started their first job. However, even though they compared youths who did or did not move directly from university to their first job, the availability of only two waves of data precludes detection of the precise shape of the self‐esteem trajectory across the school‐to‐work transition, and the reliance on a sample of college students limits the generalizability of the results to youths who do not attend college. In contrast, our six‐wave longitudinal study spanning 14 years allowed us to test different patterns of change before, during, and after the transition to work (see Figure 1) in a sample with substantial socioeconomic diversity. Furthermore, we adopted a piecewise growth curve modeling approach (Duncan et al., 2013; Kim & Kim, 2012) to model the different phases of the change process associated with the school‐to‐work transition.

The first aim of our study was to examine the potential beneficial impact of the school‐to‐work transition on youths’ self‐esteem development. We tested whether the transition to work enhances self‐esteem development for young workers after they begin their first job, accelerating the linear increase that typically occurs during late adolescence and young adulthood. The second aim of our study was to explore whether several school‐ and job‐related variables (described in the next section) predict the self‐esteem trajectory during the school‐to‐work transition.

### 1.3 The moderating role of school‐ and job‐related variables

Educational expectations (i.e., the level of education a person expects to attain) play an essential role in setting goals for the future and act as motivational forces that propel students to pursue higher educational levels (Lawson et al., 2020). Educational expectations predict students’ grades as well as their actual educational attainment (Boxer et al., 2011; Cunningham et al., 2009).

It is likely that lower educational expectations make youths less inclined to engage with school tasks and to invest less in learning and pursuing education given their lack of belief in their ability to achieve high educational goals (Schunk & DiBenedetto, 2018). Youths with low educational expectations are more likely to drop out of school (Fan & Wolters, 2014) and less likely to attend university (Suh & Suh, 2006), suggesting that they will transition into the workforce at an earlier age than those with higher educational expectations.

In addition to educational expectations, college attendance might also affect self‐esteem development. Individuals with less education are more likely to be employed as unskilled workers and experience intermittent unemployment, which may have detrimental long‐term effects on self‐esteem (Prause & Dooley, 1997). Conversely, individuals with college degrees tend to have higher status occupations and perform better in their jobs (see Roth et al., 1996), which may provide a boost to self‐esteem. Consistent with this idea, more educated individuals show higher overall self‐esteem trajectories than less educated individuals (Orth et al., 2010).

We also examined the interactive effect of educational expectations and college graduation on self‐esteem development during the school‐to‐work transition. We postulated two ways in which educational expectations and college graduation might interact. First, college attendance may only effect self‐esteem development if it matches one's educational expectations (i.e., if one expected to attend college). This scenario suggests a *congruence* effect, such that individuals who expected to attend college and, in fact, did attend college would show the steepest positive self‐esteem trajectory, whereas individuals who expected to attend college but did not do so, would experience a flat or declining self‐esteem trajectory. In a second scenario, we speculated that the opposite pattern is also plausible; that is, individuals who expected to attend college and did attend college may not increase in self‐esteem because they simply matched their expectations, whereas individuals who graduated from college despite not expecting to do so might experience a substantial boost to their self‐esteem because they surpassed their expectations. This second scenario is consistent with William James’ (1890) hypothesis that self‐esteem reflects a person's successes divided by their expectations of success (i.e., pretensions); in other words, self‐esteem derives from an individual's accomplishments tempered by what they expected to accomplish. Given that both scenarios seem plausible, we tested the interaction effect in an exploratory way.

Turning to the job‐related variables, two important factors should be considered. First, the age when an individual transitions to work is of crucial importance, given that events that occur earlier or later than expected may have a greater impact on personality than those occurring on‐time (Luhmann et al., 2014; Neugarten, 1976). In the Reitz et al. (2020) study described above, the transition to work occurred at the end of university and was thus likely to be perceived as normative by the vast majority of participants (Heckhausen et al., 2010). In contrast, the impact of non‐normative school‐to‐work transitions remains an understudied topic. Based on this reasoning, we investigated how the age when youth transition from school to work (which reflects the normative vs. non‐normative timing of the transition) affects self‐esteem change. Transition that occurred around the average age of the transition to work were considered normative, whereas transitions that occurred much before or much after the average age of the transition were considered non‐normative.^1^

Second, whether the job is permanent or temporary is an important proxy for job insecurity (De Cuyper & De Witte, 2005), which represents a powerful stressor that threatens workers' well‐being (De Cuyper & De Witte, 2005, 2006; De Witte et al., 2015). Given that a temporary contract entails uncertainty about the future of one's job, individuals may be less inclined to invest in the newly acquired social role. Consequently, we expected that youth with a temporary job would show less positive self‐esteem changes during the school‐to‐work transition compared to those with a permanent job.

### 1.4 Covariates of self‐esteem development

To examine the robustness of our results, we controlled for gender, socioeconomic status (SES), and negative life events, which are all associated with the development of self‐esteem (Erol & Orth, 2011; Orth & Robins, 2019; Orth et al., 2012; von Soest et al., 2016). Orth and Robins (2019) reviewed recent longitudinal studies and meta‐analytical findings, and found that men and high SES individuals report slightly higher levels of self‐esteem than women and low SES individuals, although the association between SES and self‐esteem does not extend to cross‐lagged effects (Kuster et al., 2013; Orth et al., 2012). Furthermore, negative life events, such as the death of a parent or serious accident or injury, have a negative impact on both the level and slope of self‐esteem (Orth & Luciano, 2015). The effect of negative life events on self‐esteem is consistent with two principles from research on personality development: (1) the *plasticity principle* (Roberts & Nickel, 2021), which states that personality traits (i.e., self‐esteem) are open systems that can be influenced by environmental factors, including life events, at any age, and (2) the so called *socialization effect* (Specht et al., 2014), which states that life experiences can contribute to personality change. Consequently, we expect that negative life events will be associated with lower levels of self‐esteem.

### 1.5 The present study

The present study examined changes in self‐esteem associated with entering the workforce using data from 368 youths assessed up to six times across a 14‐year time span. We tested several alternative models of self‐esteem change and whether the shape of the self‐esteem trajectory varied as a function of several school‐ and job‐related variables, including educational expectations, college graduation, the interaction between educational expectations and college graduation, age when the transition to work occurred, and whether the first job was temporary or permanent. In addition to these main research questions, we also tested the effects of gender, SES, and negative life events as covariates in the models.^2^

## 2 METHOD

### 2.1 Participants

Data came from a longitudinal multi‐cohort study conducted in Genzano, a community near Rome, from 1989 to 2012. Participants (*N* = 368; 59.1% female; 100% Caucasian) were recruited from public junior high schools. During the years when the study was conducted, the participating families closely matched the socioeconomic profile of Italy as a whole (Istituto Italiano di Statistica, 2002). In this study, we focused on data collected from two cohorts assessed five or six times, across a 14‐year time span from 1998 to 2012 (Wave 1 = 1998; Wave 2 = 2000; Wave 3 = 2002; Wave 4 = 2004; Wave 5 = 2008; Wave 6 = 2012), when self‐esteem was assessed. Cohort 1 (*n* = 278) was assessed at all waves, whereas Cohort 2 (*n* = 90) was added to the study at Wave 2, and consequently only participated at Waves 2 to 6.

As shown in Table 1, youth's mean age was 15.3 years (*SD* = 1.24; range = 13 to 17) at Wave 1 and 29.3 years (*SD* = 1.24; range = 27 to 31) at Wave 6. As shown in Table 1, the percentage of participants who were employed at some point during the study increased from 2% at Wave 1 to 100% at Wave 6. At Wave 1, 45.1% of unemployed youths attended the first year of high school, 30.4% the second year of high school, 19.4% the third year of high school, 3.3% fourth year of high school and 1.6% the fifth year of high school.^3^ From Waves 4 to 5, all of the unemployed youths were enrolled in university programs. All participants started a job at some point during the study.

**TABLE 1:** Descriptive statistics among key study variables

Wave	*N*	Sex		Age		Self‐esteem		Negative life events		Employment status		Educational status	
		Women	Men	*M*	*SD*	*M*	*SD*	*M*	*SD*	No %	Yes %	School %	University %
Wave 1	278	164	114	15.30	1.24	3.24	0.44	0.91	0.73	98	2	98	0
Wave 2	362	214	148	17.30	1.26	3.25	0.51	0.87	0.90	89	11	89	0
Wave 3	368	217	151	19.30	1.18	3.28	0.49	2.20	1.37	79	21	58	20
Wave 4	364	215	149	21.31	1.27	3.32	0.52	0.76	0.84	53	47	0	51
Wave 5	282	166	116	25.33	1.24	3.38	0.49	0.61	0.82	21	79	0	35
Wave 6	177	104	73	29.30	1.24	3.40	0.43	0.53	0.55	0	100	0	24

### 2.2 Procedure

At Wave 1, youths completed a questionnaire administered in classrooms by two trained researchers who provided instructions and explained that the responses to the questionnaires would be kept confidential. In addition, parent consent and youth assent were obtained, as well as the approval of the school districts. After youths ended their compulsory education (depending on the age of the youth, this occurred as early as Wave 2 or as late as Wave 6), they completed the questionnaire and consent form by mail. At each wave, participants received a payment of €25 (about US$35), or an equivalent dinner voucher, for completing the questionnaire.

### 2.3 Attrition

For Cohort 1, the percentage of missing data was approximately 2% at Wave 2, 0% at Wave 3, 1% at Wave 4, 31% at Wave 5, and 59% at Wave 6. For Cohort 2, the percentage of missing data was 0% at Wave 3, 1% at Wave 4, 0% at Wave 5, 30% at Wave 6. The attrition was caused primarily by the researchers’ inability to contact participants who moved out of the area. Importantly, the Missing Completely at Random (MCAR) Little's test (Enders, 2010) supported the MAR hypothesis [*χ* ^2^(44) = 52.241, *p* = .184], suggesting no bias due to missingness in our data. In addition, to investigate any potential impact of missing data on our analyses, we compared youths who participated in all assessments with those who dropped out of the study, on study variables assessed during the first wave in which they participated (i.e., Wave 1 for Cohort 1 and Wave 2 for Cohort 2). Youths who dropped out were significantly more likely to be younger (*Ms* = 15.05 vs. 15.84 years, *d* = −0.67), come from lower SES families (*d* = −0.23), and have lower educational expectations (*Ms* = 3.97 vs. 4.28, *d* = −0.20); no significant differences were found for self‐esteem, employment status, gender, college graduation, or negative life events. Given that these differences were small or nonsignificant (except age), and given that the MCAR test indicated the absence of systematic attrition, we did not consider attrition as a serious concern in the present study. Accordingly, in all of our analyses we used Maximum Likelihood estimation for dealing with missing data (Enders, 2010) and, as explained below, we also included variables that presented significant differences between youths who participated in all assessments and those who dropped out as covariates to control for in our analyses.

### 2.4 Measures

#### 2.4.1 Self‐esteem

Self‐esteem was assessed with the 10‐item Rosenberg Self‐Esteem Scale (RSE; Rosenberg, 1965), one of the most widely used scales for measuring global self‐esteem (Robins et al., 2001). The response format was a 4‐point scale ranging from 1 (“strongly disagree”) to 4 (“strongly agree”). Reliability coefficients were: *α* = 0.85 and *ω* = 0.88 at Wave 1; *α* = 0.85 and *ω* = 0.89 at Wave 2; *α* = 0.86 and *ω* = 0.90 at Wave 3; *α* = 0.87 and *ω* = 0.91 at Wave 4; *α* = 0.89 and *ω* = 0.92 at Wave 5; *α* = 0.90 and *ω* = 0.93 at Wave 6.

To facilitate interpretation of the results and the plotted trajectories, we transformed the self‐esteem scores into T scores (with mean equal to 50 and standard deviation equal to 10 T score points), using the grand mean and standard deviation of the overall sample across all assessments.

#### 2.4.2 Educational expectations

At the first wave for each cohort, participants were asked, “What level of education do you think you will actually achieve?”, and provided with the following response options: “Attend at least a few years of middle school” (=1); “Graduate from trade school” (=2); “Graduate from high school” (=3); “Obtain a professional certification” (=4); “Attend at least a few years of university” (=5); “Graduate from university” (=6). The mean for educational expectations was 4.06 (*SD* = 1.55). This item was converted into *z* scores.

#### 2.4.3 College graduation

We created a dichotomous variable contrasting youths who did (=1) or did not (=0) earn a college degree prior to starting their first job. One hundred and one youth (27.3%) graduated from college.

#### 2.4.4 Age at first job

This variable represented the age when the participants reported starting their first job (*M* = 22.65 years; *SD* = 3.63). This variable was converted into *z* scores.

#### 2.4.5 Type of job contract

We created a dichotomous variable contrasting youths with a temporary (=0) versus permanent (=1) contract for their first job. One hundred and fifty three youths (41.6%) had a temporary contract.

#### 2.4.6 Socioeconomic status (SES)

SES was based on a composite of five items: mother's highest level of education completed, father's highest level of education completed, mother's job, father's job (ranked from higher to lower on the basis of their reported annual income), and annual family income. The SES variable was estimated as the first unrotated component in a Principal Component Analysis of these five items. This was a formative indicator and thus, we do not report alpha reliability for SES. SES was assessed at the first available wave for each cohort.

#### 2.4.7 Negative life events

At each wave, youths reported whether or not they experienced nine negative life events during the past year, including “divorce of parents”, “death of a parent or stepparent”, “death of a close relative or friend”, “victimization by serious physical attack or assault”, “serious illness”. This variable was computed as the sum of all nine events, and thus ranged from 0 (no events experienced) to 9 (all events experienced). This was a formative indicator and thus, we do not report any reliability estimates.

### 2.5 Plan of analysis

As measurement invariance across time is essential for latent growth curve models, we tested the level of longitudinal invariance exhibited by our self‐esteem measure (Widaman et al., 2010). Specifically, we built a set of measurement models including, at each wave: (1) self‐esteem factors and (2) two method factors that accounted for bias due to positive and negative keying of the items (Alessandri et al., 2015). The self‐esteem factors and the positive and negative wording factors were correlated across waves, but positive wording factors were uncorrelated with negative wording factors, and all wording factors were uncorrelated with the self‐esteem factors. Also, the measurement models included longitudinal correlations between the same items measured at different waves (Cole & Maxwell, 2003). Including these correlations controls for possible bias due to indicator‐specific variance that is not captured by the self‐esteem and wording factors. We first assessed configural invariance (Widaman et al., 2010) by fixing the mean and the variance of the self‐esteem latent factors to 0 and 1 respectively across waves, and all variances of the method factors to 1, while allowing the loadings and the intercepts at each wave to be freely estimated. In two subsequent models, we tested metric and strict invariance by progressively constraining the loadings and intercepts of indicators, respectively, to be equal across waves. We compared the fit of these three models to determine which model to use in the subsequent analyses of self‐esteem change.

#### 2.5.1 Unconditional piecewise growth curve models

We examined the appropriate pattern of change for self‐esteem during the transition to work by subdividing the change process into three distinct moments: (1) before the school‐to‐work transition; (2) after the school‐to‐work the transition; and (3) a sudden change occurring at the moment of the school‐to‐work transition. In doing so, we implemented a series of piecewise growth curve models (Kim & Kim, 2012), in which we estimated an increasing number of growth factors and parameters. In these piecewise models, the metric of time was centered around the age of the school‐to‐work transition; that is, for each participant, his or her age at each assessment was centered on the year in which he or she started a job. This procedure allowed us to test the short‐ and long‐term effects of the transition to work on the self‐esteem trajectory. In these models, the intercept represents the estimated self‐esteem level during the year when the transition to work occurred, the first linear slope represents change occurring before the transition to work, the second linear slope represents change occurring after the transition to work (i.e., the second linear slope), and a fourth parameter, the boost, represents sudden change occurring at the time of the transition to work.

The first two models in Figure 1 tested whether single‐phase latent growth curve models fit the data. Panel A shows an intercept‐only model, in which only a latent intercept was estimated, whereas Panel B shows a model with both an intercept and a single linear slope. By implementing these two models, we tested whether the transition to work had an impact on youths’ self‐esteem development. The other three models shown in Figure 1 tested which kind of effect the transition to work exerted on self‐esteem development. Specifically, Panel C tests for a “boost” effect (i.e., sudden change only); Panel D tests for gradual change only; and Panel E tests for both a possible boost and a gradual change occurring after the transition to work.

#### 2.5.2 Conditional piecewise growth curve models

Once the best‐fitting unconditional model was identified, we moved to testing the effects of educational expectations, college graduation, the interaction between educational expectations and graduation, age at first job, type of contract, and the covariates (sex, SES, and negative events), adding each variable to the models as a time‐invariant covariate (TICs), except for the negative life events scale, which was added to the model as a time‐varying covariate (TVC) (Mehta & West, 2000; Preacher et al., 2008). Importantly, not every growth parameter was regressed on each predictor. In fact, we assumed that only educational expectations, measured at the initial stage of the study for both cohorts, would impact every phase of the changing process. We treated graduation, age of first job, type of contract and their interactions, as variables concerning only the transition to work. For this reason, we assumed that they impact self‐esteem development during and after the school‐to‐work transition, and not before the school‐to‐work transition. At the same time, we treated the covariates as structural variables that could impact every phase of the changing process. Thus, in our models only the intercept and the linear slope after the transition were regressed on the moderating variables, while every growth factor was regressed on educational expectations and the covariates.

In testing the conditional models, we proceeded as follows. First, we tested the effect of the covariates adding all of them to the model simultaneously. After that, we specified a single model for each moderating variable, examining their effects separately from the others but maintaining the covariates. Specifically, we started with testing the effect of educational expectations on the intercept and on the first and the second linear slopes, representing change before and after the school‐to‐work transition, respectively. We followed the same procedure for the other moderating variables, but only allowing the intercept and the slope representing change after the school‐to‐work transition to regress on the moderators. Finally, we tested a model with all of the variables, including both the moderators and the covariates, along with the interaction term between educational expectations and college graduation. Notably, in testing the effect of this interaction, both of the variables and the interaction term were added to the model simultaneously.

### 2.6 Statistical analysis

For estimating growth models and for handling missing data, we used M*plus* 8.3 with Full Information Maximum Likelihood (FIML) estimation (Muthén & Muthén, 2017), which produces more reliable and less biased results compared to listwise or pairwise deletion (Schafer & Graham, 2002). For measurement invariance models, goodness of fit was evaluated by inspecting the chi‐square statistic (*χ^2^*), the Comparative Fit Index (CFI) and the Root Mean Square Error of Approximation (RMSEA). Values of CFI > 0.90 and RMSEA < 0.08 were considered acceptable (Kline, 2016). Differences among nested models were judged significant in the presence of changes in CFI (i.e., ΔCFI) greater than 0.01 (Cheung & Rensvold, 2002; Schmitt & Kuljanin, 2008).

The fit of all piecewise growth curve models was assessed using the Bayesian information criterion (BIC). When comparing models using BIC, lower values indicate better model fit (Burnham & Anderson, 2004). Accordingly, models were compared by subtracting the BIC of each competing model from the BIC of the baseline model. Following suggestions by Burnham and Anderson (2004), if the BIC difference between a competing and baseline model is less than 2, the competing model is considered the best fitting one; if the difference lies between 4≤ and ≤7 there is considerably less support for the competing model; whereas models with a difference >10 have essentially no support and the baseline model remains the reference model to be compared with other competing models. Finally, considering the large number of statistical tests conducted, and the fact that our hypotheses were not pre‐registered, we used *p* < .01 as the threshold for interpreting significant results.

## 3 RESULTS

Table 1 shows descriptive information for the primary study variables. Intercorrelations among all study variable (see Table 2) showed moderate to high test‐retest correlations for self‐esteem, demonstrating high rank‐order stability across two‐ and four‐year time intervals. It is noteworthy that educational expectations were positively correlated with SES (*r* = 0.38), graduation (*r* = 0.28), and age of first job (*r* = 0.28), whereas graduation was positively correlated with age of first job (*r* = 0.49). Negative life events were generally negatively correlated with self‐esteem across waves. Results of the measurement invariance analyses are presented in Table 3. The fit of the configural, metric, and scalar invariance models were all acceptable, and did not differ significantly from each other, suggesting that scalar invariance was reached.

**TABLE 2:** Zero‐order correlations among study variables

	(1)	(2)	(3)	(4)	(5)	(6)	(7)	(8)	(9)	(10)	(11)	(12)	(13)	(14)	(15)	(16)	(17)
1. Self‐esteem (W1)	–																
2. Self‐esteem (W2)	0.58**	–															
3. Self‐esteem (W3)	0.53**	0.67**	–														
4. Self‐esteem (W4)	0.50**	0.56**	0.64**	–													
5. Self‐esteem (W5)	0.44**	0.45**	0.54**	0.57**	–												
6. Self‐esteem (W6)	0.27**	0.46**	0.47**	0.57**	0.61**	–											
7. Sex	0.14**	0.15**	0.09	0.03	0.03	−0.03	–										
8. SES	0.10	0.06	0.02	0.08	0.06	0.02	−0.01	–									
9. Educational Expectations	0.21**	0.09	0.17**	0.18**	0.13*	0.10**	−0.13*	0.38**	–								
10. Graduation	0.05	0.08	0.03	0.12*	0.11*	0.12**	−0.13*	0.28**	0.28**	–							
11. Age at First Job	0.07	0.04	0.02	0.05	0.05	0.02	−0.05	0.17**	0.28**	0.49**	–						
12. Type of Contract	0.03	−0.03	0.03	0.03	0.05	0.09	0.03	−0.02	−0.06	−0.11*	−0.03	–					
13. Negative Events (W1)	−0.30**	−0.22**	−0.23	−0.26**	−0.17**	−0.26**	−0.06	−0.08	−0.13*	−0.06	−0.07	−0.02	–				
14. Negative Events (W2)	−0.20**	−0.16**	−0.16	−0.24**	−0.17**	−0.13	−0.01	−0.07	−0.21**	−0.11*	−0.22**	0.10*	0.34**	–			
15. Negative Events (W3)	−0.07	−0.12*	−0.10	−0.13*	−0.15**	−0.16**	0.11*	−0.08	−0.19**	−0.03	−0.17**	0.08	0.14**	0.37**	–		
16. Negative Events (W4)	−0.19**	−0.22**	−0.31	−0.42**	−0.29**	−0.32**	−0.05	−0.06	−0.14**	−0.12*	−0.19**	−0.04	0.28**	0.36**	0.31**	–	
17. Negative Events (W5)	−0.22**	−0.19**	−0.23	−0.36**	−0.40**	−0.35**	−0.09	−0.01	−0.11*	−0.13*	−0.11*	−0.01	0.20**	0.25**	0.30**	0.53**	–
18. Negative Events (W6)	−0.28**	−0.31**	−0.30	−0.38**	−0.31**	−0.41**	−0.13*	0.00	−0.14**	−0.16**	−0.09	−0.04	0.47**	0.42**	0.29**	0.44**	0.56**

* *p* < .05

** *p* < .01

**TABLE 3:** Longitudinal measurement invariance of rosenberg self‐esteem scale across six waves

Model	*χ* ^2^	*df*	CFI	RMSEA	CI 90%	ΔCFI
Configural	2043.37**	1419	0.937	0.034^+^	[0.031, 0.037]	–
Metric	2139.71**	1469	0.933	0.035^+^	[0.031, 0.038]	−0.004
Scalar	2273.32**	1519	0.924	0.036^+^	[0.033, 0.039]	−0.009

Abbreviations: CFI, comparative fit index; CI 90% = RMSEA confidence interval 90%; *df*, degree of freedom; RMSEA = root mean square error of approximation; χ^2^, chi‐square statistic; ΔCFI, CFI difference.

^n.s.^ *p* > .05;

* *p* < .05

** *p* < .01

### 3.1 Unconditional piecewise growth curve models

The model with a linear increase before the school‐to‐work transition and a gradual change after the school‐to‐work transition (Figure 1, Panel D) had the best fit to the data, according to the BIC (Table 4). This model included an intercept, as well as two slopes representing the phases of change occurring before and after the school‐to‐transition. Indeed, this model (BIC = 15,483.787) fit the data significantly better than the no growth model (BIC = 15,608.774; ΔBIC = |124.987|), the linear slope model (BIC = 15,502.183; ΔBIC = |18.396|), the model including the intercept, one slope and a boost during the school‐to‐work transition (BIC = 15,512.291; ΔBIC = |28.504|) and the model with intercept, boost and gradual change (BIC = 15,503.067; ΔBIC = |19.280|). Therefore, we used the gradual change model in all subsequent analyses.

**TABLE 4:** BIC of all the unconditional and conditional models

Model	BIC
*Unconditional models*	
Intercept‐only model (Figure 1‐ Panel A)	15,608.774
Intercept and a single linear slope model (Figure 1‐ Panel B)	15,502.183
Sudden change only model (Figure 1‐ Panel C)	15,512.291
Gradual change only model (Figure 1‐ Panel D)	15,483.787
Sudden and gradual change model (Figure 1‐ Panel F)	15,503.067
*Conditional models*	
Only covariates model (Model 1)	15,459.411
Educational expectations predictor model (Model 2)	15,457.120
Graduation predictor model (Model 3)	15,464.250
Age first job predictor model (Model 4)	15,463.939
Type of job predictor model (Model 5)	15,468.284
All covariates and predictors model (Model 6)	15,486.284

Note

BIC = Bayesian Information Criterion.

**FIGURE 2:**
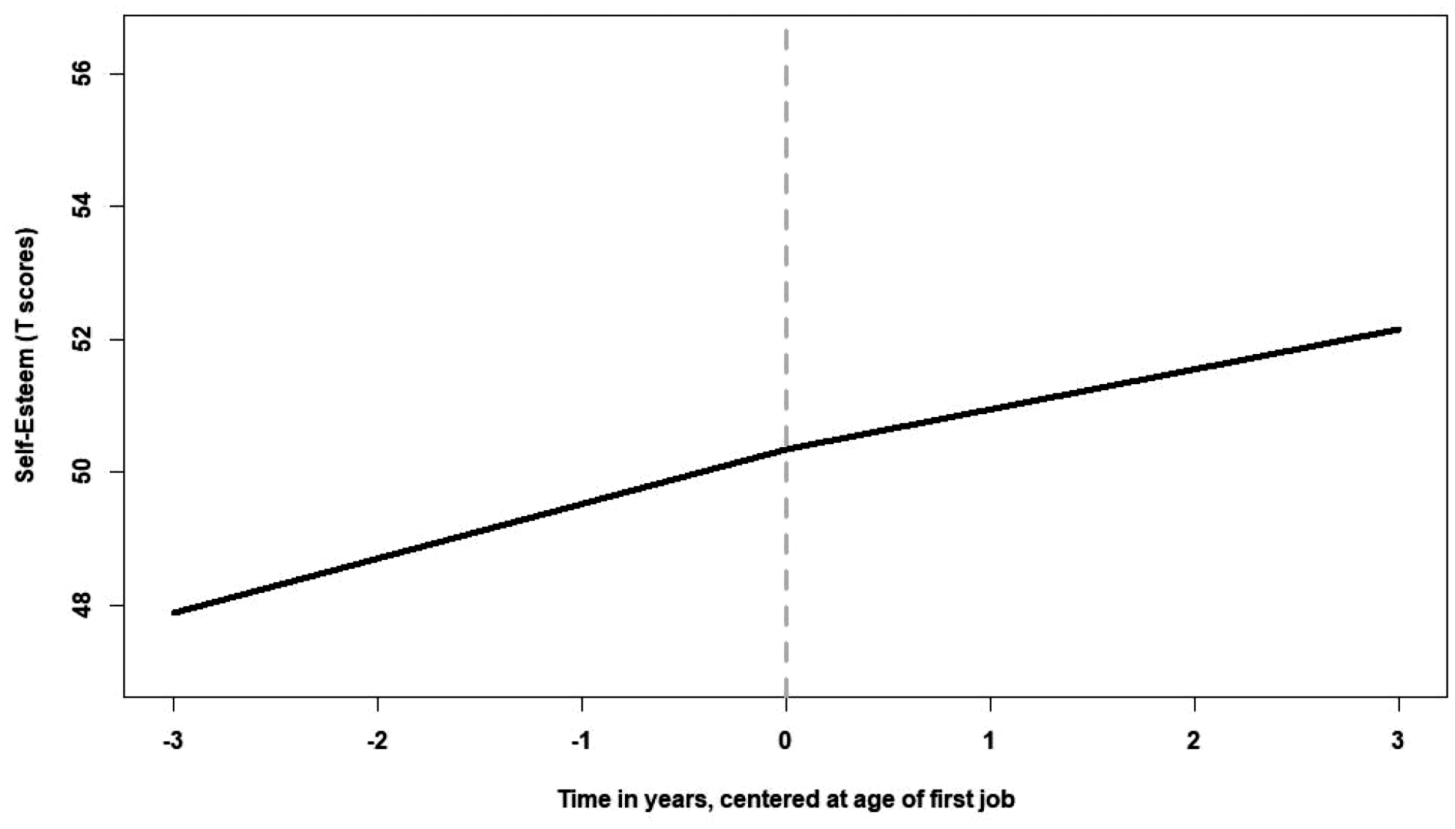
Estimated unconditional change for self‐esteem. The grey dotted vertical line represents the moment of transition to work. The solid lines represent changes in self‐esteem before and after the transition to work

Figure 2 shows the trajectory estimated from the unconditional model. The intercept mean (50.355, *z* = 99.019, *p* < .001) and variance (74.518, *z* = 10.433, *p* < .001), the first slope mean (0.25, *z* = 4.002, *p* < .001) and variance for change before the school‐to‐work transition (0.34, *z* = 3.714, *p* < .001), and the second slope mean (0.18, *z* = 2.977, *p* < .001) and variance for change after the school‐to‐work transition (0.44, *z* = 4.275, *p* < .001), were all significant. These parameter estimates indicated that self‐esteem increased during the entire study period, but the increase was more rapid before (slope = +0.25) versus after (slope = +0.18) the school‐to‐work transition. However, the difference between the two slopes was minimal and a model with these slopes constrained to equality (BIC = 15,478.333) fit the data slightly better than the model with freely estimated slopes (BIC = 15,483.787; ΔBIC = |5.454|). The variance of the slope after the transition (0.44) was higher than the variance of the slope before the transition (0.34), suggesting that the transition to work increased variability in self‐esteem change. Finally, the intercept was positively correlated with the slope before the transition (0.49, *p* < .001), and negatively correlated with the slope after the transition (−0.55, *p* < .001); this indicates that youths with higher levels of self‐esteem increased more prior to the school‐to‐work transition but increased less after the school‐to‐work transition.

### 3.2 Effect of covariates

When tested alone in the first model, all of the covariates showed null effects. However, when tested in the final model, along with all the other variables, two significant results emerged. First, men reported higher self‐esteem than women before the transition to work. Second, negative life events were negatively associated with momentary self‐esteem scores across all models (*M_randomslope_* = −0.748, *SD_randomslope_* = 0.016). SES did not predict change in self‐esteem in any model.

### 3.3 Conditional piecewise growth curve models

Table 5 presents parameter estimates from models testing the effects of the hypothesized predictors of self‐esteem change (see Table 4 for the BIC of all the tested conditional models). The first set of models included the school‐related predictors. The individual effects of educational expectations and graduation (Model 2 and 3; see Table 5), showed an intricate pattern of interaction in predicting the growth factors. Considered alone, both educational expectations (Model 2) and graduation (Model 3) were significant predictors of the intercept but not the slopes, meaning that higher levels of educational expectations and achieving graduation were associated with a higher self‐esteem levels at the time of graduation.

**TABLE 5:** Effects of educational expectations, graduation, age at first job, type of job, and covariates on self‐esteem trajectories

Estimates	Basic model	Conditional models					
		Model 1	Model 2	Model 3	Model 4	Model 5	Model 6
Means and variances of growth curve factors^a^							
*Means*							
Intercept	50.355**	51.094**	50.942**	50.362**	51.062	51.056**	50.444**
Slope before transition	0.247**	0.391**	0.418**	0.403**	0.424**	0.391**	0.441**
Slope after transition	0.181**	0.162*	0.170*	0.168	0.184*	0.097	0.103
Random slope Neg. Ev.		−0.768**	−0.750**	−0.751**	−0.739**	−0.766**	−0.722**
*Variances*							
Intercept	74.518**	64.951**	63.555**	63.673**	64.500**	65.017**	62.929**
Slope before transition	0.345**	0.248**	0.241**	0.243**	0.245**	0.246**	0.223**
Slope after transition	0.444**	0.437**	0.434**	0.433**	0.440**	0.442**	0.423**
Random Slope Neg. Ev.		2.503**	2.468**	2.512**	2.551**	2.497**	2.475**
Regression coefficients of predictors and covariates of growth curve factors							
*Predicting intercept*							
Sex (0 = male, 1 = female)		0.483	0.844	0.750	0.585	0.498	1.000
SES		0.248	−0.306	−0.040	0.057	0.244	−0.469
Ed. Exp.			1.405**				0.969^+^
Graduation (0 = no, 1 = yes)				2.240**			−2.487
Ed. Exp. × Graduation							0.742
Age First Job					0.464**		0.306
Type of job (0 = temporary, 1 = permanent)						0.127	0.404
*Predicting slope before transition*							
Sex (0 = male, 1 = female)		−0.348**	−0.366**	−0.344**	−0.342**	−0.346**	−0.364**
SES		−0.080	−0.047	−0.081	−0.085	−0.080	−0.049
Ed. Exp.			−0.107^+^				−0.100
*Predicting slope after transition*							
Sex (0 = male, 1 = female)		−0.106	−0.131	−0.099	−0.106	−0.120	−0.133
SES		−0.001	0.039	0.000	0.007	0.006	0.037
Ed. Exp.			−0.092				−0.043
Graduation (0 = no, 1 = yes)				0.038			1.783**
Ed. Exp. × Graduation							−0.340**
Age First Job					−0.008		−0.004
Type of Job (0 = temporary, 1 = permanent)						0.226	0.254

Note

M*plus* provides only unstandardized estimates for the present analyses, however we standardized self‐esteem scores before conducting analyses. Thus, these estimates can be interpreted as standardized effects. Neg. Ev. = negative life events; Ed. Ex. = educational expectations.

^a^ In the conditional models, means are intercepts and variances are residual variances.

^+^ *p* < .10;

* *p* < .05

** *p* < .01

The second set of models tested the job‐related variables. These models (Model 4 and 5) revealed a small association of age at first job with the intercept, and no significant association of type of contract on self‐esteem change occurring during the school‐to‐work transition. Accordingly, only age at first job was positively associated with higher overall levels of self‐esteem trajectories. Neither job‐related variable was associated with the slope parameters.

**FIGURE 3:**
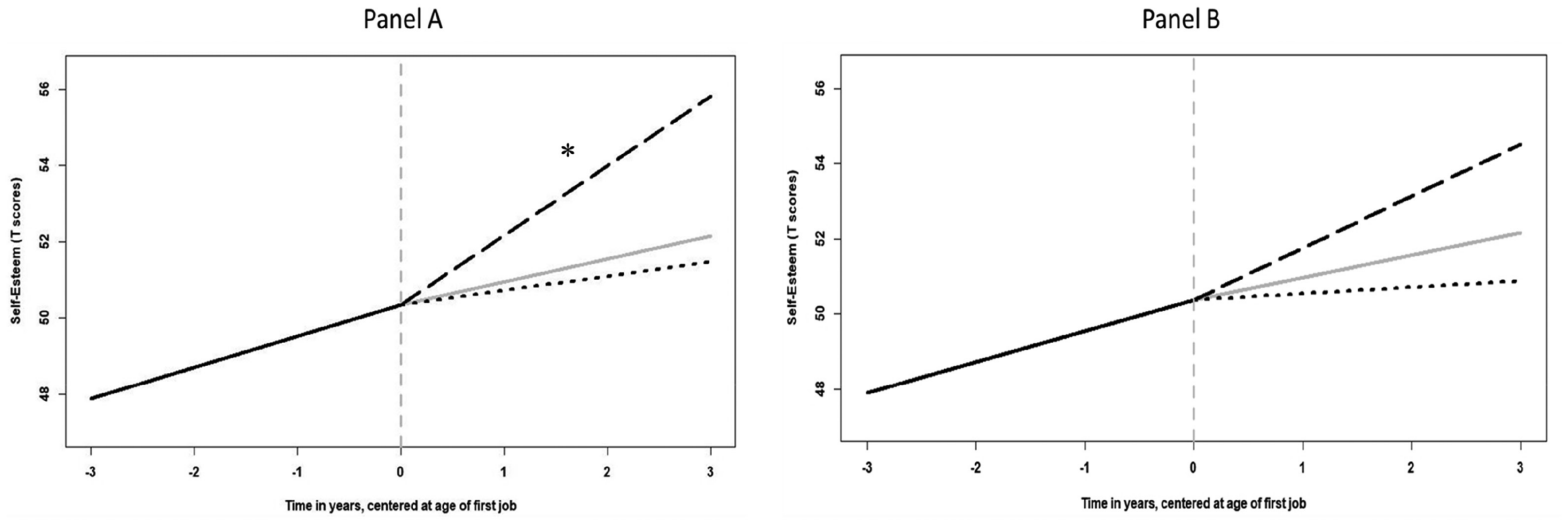
Estimated conditional change for self‐esteem. Panel A represents trajectories for youths with low educational expectations, while Panel B represents trajectories for youths with high educational expectation. The grey dotted vertical lines represent the moment of transition to work. The grey solid lines after the transition to work represents the unconditional average trajectory. The black dashed lines represent the estimated self‐esteem change after the transition to work for college graduates (upper line). The black dotted line represents self‐esteem change estimated for non‐graduates (lower line). High and low levels of educational expectations were operationalized as 1 standard deviation above and below the mean, respectively. The asterisk indicates the significant mean slope

Finally, the last model (Model 6) included all of the predictors and covariates. This model allowed us to test whether the above results remained significant even after adjusting the effects of all of the variables simultaneously. The interaction between educational expectations and college graduation had a significant effect on the slope after the transition to work. To understand this interaction, Figure 3 shows how youth with low (Panel A) and high (Panel B) educational expectations changed in self‐esteem when they did versus did not graduate from college prior to beginning their first job. Among youths with low educational expectations (Panel A), self‐esteem continued to significantly increase after the transition to work for college graduates (2.269, *z* = 2.529, *p* < .05), but not for non‐college graduates (0.146, *z* = 0.224, *p* = .822). Conversely, among youth with high educational expectations (Panel B), self‐esteem did not increase significantly for either college graduates (1.503, *z* = 1.651, *p* = .099) or non‐college graduates (0.060, *z* = 0.092, *p* = .926).

## 4 DISCUSSION

Self‐esteem is often considered one of the key psychological characteristics associated with success and well‐being (Orth & Robins, In press). Consequently, identifying predictors of self‐esteem change, especially during important life transitions, is of great importance to improve positive outcomes for individuals and society. Sources of self‐esteem development are not only internal or biological (Kendler et al., 1998), but in large part environmental in nature (Neiss et al., 2006, 2009). Based on a role transition perspective, and guided by social investment and sociometer theories, the present study investigated changes in self‐esteem trajectories during the school‐to‐work transition. We used cohort‐sequential longitudinal data from 368 Italian youths followed over 14 years, spanning the developmental period from age 13 to 31.

Our results suggested the presence of a two‐phase pattern of change for self‐esteem: one before and the other after youths’ transition from school to work. These trajectories were both linear and positive, but slightly differed in their rate of change, with steeper increases in self‐esteem observed before the transition to work and more gradual increases observed after the transition. These patterns of change were predicted by several moderators and covariates, including educational expectations, college graduation, and gender. Below, we discuss each of these results in detail.

### 4.1 Self‐esteem changes during the school‐to‐work transition

Our data suggest that self‐esteem changes differentiated into two different phases, with a slightly more rapid increase in self‐esteem before versus after the school‐to‐work transition. Thus, the two average trajectories differed in steepness, but not in direction, consistent with previous research showing a positive developmental trajectory from late adolescence through young adulthood (Orth et al., 2018). However, given that differences between BICs associated with the freely estimated slopes model and the fixed slopes model lie in the 4 to 7 range (5.5), the evidence supporting differences between the pre‐ and post‐work transition slopes is not compelling and needs to be replicated. Furthermore, the significant slope variance before and after the transition, and the greater variance after the transition, corroborate Hutteman et al.’s (2014) point that people differ in the way they deal with developmental tasks associated with important life transitions, such as the transition to fulltime employment. These differences can be understood not only on the basis of their individual differences but also in light of personal relevance of the event. Indeed, it is likely that in our sample, many participants started a job that did not perfectly align with their personal expectations, aspirations, or qualifications (or even by necessity), and this reduced their personal investment in the job.

Nonetheless, it is noteworthy that among the models that tested different patterns of self‐esteem change (see Figure 1), the model with change in the slope before versus after the transition (Panel D) fit better than the model with no change in the slope before versus after the transition (Panel B). However, follow‐up analyses did not provide unequivocal evidence that the two slopes differ significantly from each other. Consequently, more research is needed to confirm whether or not the self‐esteem trajectory changes before versus after the transition to work. Regardless, we found no evidence for our hypothesis that entering the job market would provide a substantial boost to the self‐esteem trajectory; if there is a difference, it is in the opposite of the predicted direction, that is, self‐esteem shows more substantial increases *before* the transition to work. It is likely that rather than providing a general boost to self‐esteem for all individuals, the transition to work increases self‐esteem for some people but decreases it for others, leading to greater variability in the individual slopes after versus before the transition. What remains to be clarified is whether these findings are due to historical factors (e.g., the economic recession made it more difficult for individuals to find their ideal job) or reflect a more general effect linked to work socialization processes.

Another possibility is that starting a new job had only a marginal effect beyond other structural factors impacting youths’ development. Indeed, according to Wagner et al. (2013), starting a job is only one of several life events associated with changes in self‐esteem in young adulthood. For example, one may speculate that, for many youths, potential changes caused by starting their first job were offset by contemporaneous changes in other important domains of life (e.g., social and romantic relationship, living or not living at home) that had a countervailing effect. Thus, it seems important that future studies examine how changes occurring in multiple life contexts collectively impact self‐esteem, in order to ascertain how they accumulate, amplify, or cancel out each other.

Among potential predictors of different self‐esteem patterns of change, our results suggest that the school‐related variables were the most relevant. In particular, college graduation enhanced self‐esteem change after the transition to work, corroborating prior research showing that academic achievement engenders higher self‐esteem (Chung et al., 2014; Zheng et al., 2020). To be sure, college graduation exerted a positive effect on self‐esteem change after the transition to work primarily for youths with low educational expectations. This result supports the idea that graduation leads to a gain in perceived personal value for these youths. It is likely that these effects are linked to basic components of general self‐esteem, and in particular to the sense of competence that is central to the construct (Tafarodi & Swann, 1995). Future studies should examine this possibility in more depth.

Our results suggest that the transition to work does not provide any boost to the self‐worth of non‐college graduates. Thus, entering the workforce does not necessarily enhance (or diminish) a young adult's self‐esteem, but rather it depends on the individual and his/her particular circumstances. For example, compared to college graduates, non‐college graduates typically have jobs with less autonomy and fewer opportunities to experience a sense of competence and mastery, have fewer possibilities to obtain a high‐status position in the future, and are consequently less likely to be high regarded by others, given the high value that society places on college graduation and high‐level educational achievements (see Brown & Hesketh, 2004). In contrast, college graduates often have access to highly skilled and high‐status jobs, with more financial and social gain, which, in turn, provides more opportunities to enhance their self‐worth.

Finally, neither of the two job‐related variables –the age when individuals began their first job and whether the job was permanent or temporary – affected self‐esteem change during the transition to work. Although age at first job age was positively associated with the overall level of the self‐esteem trajectory, this effect became non‐significant when other variables were added to the model. However, given the very limited range of job‐related variables examined in the present study, we are hesitant to draw any conclusions about whether other aspects of the job (e.g., the level of autonomy, status, power, stress, etc.) might predict self‐esteem change.

### 4.2 Effects of the covariates

Consistent with past research (Orth & Robins, 2019), we found that men enter the workforce with a slight self‐esteem advantage over women. In contrast, SES had no effect on either the level or the slope of self‐esteem. This is consistent with research suggesting that SES does not prospectively predict self‐esteem (Kuster et al., 2013; Orth et al., 2012). Finally, negative life events were associated with lower self‐esteem at each time point, consistent with the plasticity principle (Roberts & Nickel, 2021) and the socialization effect (Specht et al., 2014), as well as past research showing a negative effect of stressful life events on self‐esteem (Orth & Luciano, 2015; but see Orth et al., 2009).

### 4.3 Limitations and future directions

The present study has several limitations that merit attention. First, although self‐esteem has been traditionally assessed via self‐report, the exclusive reliance on self‐report data exposes our results to social desirability and shared method variance bias. Second, although our sample was very heterogeneous in terms of SES, all participants were from a single country. Thus, our results need to be replicated in different countries and cultural contexts. Furthermore, although the attrition was not systematic overall, there was a tendency for participants who dropped out to be younger, to belong to a slightly lower social class, and to hold lower academic expectations. Thus, sample composition should be taken into account to accurately interpret our results. Third, all of the participants started a job during the years of the study. Future research should examine changes in self‐esteem for unemployed youth or the so‐called NEET (i.e. “Neither in Employment nor in Education or Training”), who may find a job many years after they have left school. It is possible that prolonged unemployment has a detrimental effect on youths’ self‐esteem (Waters & Moore, 2002). A final consideration concerns the years in which the data were collected; almost all data were collected before the worldwide economic crisis that began in late 2007. The current post‐crisis working conditions do not allow today's youths to find work as easily (Scales et al., 2016). It is possible that the current economic conditions will contribute to more varied self‐esteem trajectories, as some youth obtain high status jobs while others remain chronically unemployed. Additional research on this topic is needed.

## 5 CONCLUSION

The stage of life from adolescence to young adulthood is a critical transition point in an individual's development characterized by profound socioemotional and biological changes that lay the groundwork for later adult life (Arnett, 2000; Erikson, 1968; George, 1993; Scales et al., 2016). The school‐to‐work transition is generally considered a major event in the life of youths (Bleidorn & Denissen, 2021; Bleidorn et al., 2018) that often involves significant changes in role status and how youths view themselves. The transition to work has the potential to enhance the normative development of self‐esteem, but its salutary effect depends on several factors such as youths’ educational expectations and educational attainment. A goal for the future research is to identify the factors that facilitate a successful transition to work in order to foster positive self‐worth in youths.

## CONFLICT OF INTEREST

The authors declared no potential conflicts of interest with respect to the research, authorship, and/or publication of this article.

## ETHICS STATEMENT

All data were collected in a manner consistent with ethical standards for the treatment of human subjects.

## AUTHOR CONTRIBUTIONS

Lorenzo Filosa and Guido Alessandri conceptualized the study and wrote the paper. Lorenzo Filosa ran the analyses and made all graphs, while Guido Alessandri supervised all the analyses. Richard Robins read, commented and reviewed the first draft and the subsequent revisions of the paper. Concetta Pastorelli provided data and commented the first draft of the paper.

## ENDNOTES

^1^
Notably, the average age of first job in our sample (*M* = 22.65 years) matched the average age of the Italian population (*M* = 22 years; Istituto Italiano di Statistica, 2018).

^2^
Further details on procedure and syntax are available at https://osf.io/h8b6k/?view_only=e64cf042d77344efacd075c02bdfa1f3. Data will be made available upon reasonable request to the corresponding authors. The present study was not preregistered.

^3^
Note that high school in Italy is normally 5 years long.
